# Vocal production learning in the pale spear-nosed bat, *Phyllostomus discolor*

**DOI:** 10.1098/rsbl.2019.0928

**Published:** 2020-04-15

**Authors:** Ella Z. Lattenkamp, Sonja C. Vernes, Lutz Wiegrebe

**Affiliations:** 1 Neurogenetics of Vocal Communication Group, Max Planck Institute for Psycholinguistics, Nijmegen, The Netherlands; 2 Division of Neurobiology, Ludwig-Maximilians University Munich, Germany; 3 Donders Institute for Brain, Cognition and Behaviour, Nijmegen, The Netherlands

**Keywords:** bat vocalization, pitch adjustment, vocal production learning

## Abstract

Vocal production learning (VPL), or the ability to modify vocalizations through the imitation of sounds, is a rare trait in the animal kingdom. While humans are exceptional vocal learners, few other mammalian species share this trait. Owing to their singular ecology and lifestyle, bats are highly specialized for the precise emission and reception of acoustic signals. This specialization makes them ideal candidates for the study of vocal learning, and several bat species have previously shown evidence supportive of vocal learning. Here we use a sophisticated automated set-up and a contingency training paradigm to explore the vocal learning capacity of pale spear-nosed bats. We show that these bats are capable of directional change of the fundamental frequency of their calls according to an auditory target. With this study, we further highlight the importance of bats for the study of vocal learning and provide evidence for the VPL capacity of the pale spear-nosed bat.

## 1. Introduction

Bats are highly specialized in the use of their auditory system, which allows them not only to orientate in the dark, but also to discriminate prey and surface structures and identify conspecifics with a high temporal and spectral resolution [1–7]. Bats have been shown to adjust several parameters of their echolocation and social calls in response to their social environment [8–12], which is thought to support group cohesion [6,13,14] and individual recognition [15]. Recently bats have also attracted increased attention owing to their capacity for vocal production learning (VPL), defined as the capacity to modify vocalizations ‘in form as a result of experience with those of other individuals' [16, p. 59]. VPL is related to vocal plasticity in that a change from baseline vocal parameters must occur; however, it goes beyond plasticity as it involves acoustic perception to induce a learned change towards or away from a target sound [16]. VPL can have different degrees of complexity [17,18]. While gradual vocal parameter changes towards an acoustic target have been described as a limited form of VPL, the acquisition of artificial or heterospecific vocalizations is described as complex VPL [17,19]. VPL is also distinct from vocal usage learning, which involves learning to use vocalizations in new contexts, regardless of whether they are learned or innate [16,19].

Our previous research showed that pale spear-nosed bats have volitional control over their vocalizations and possess vocal plasticity allowing them to adjust temporal and amplitude parameters of their vocalizations in a context-specific task [20]. Here, we take these experiments further and test the bats' ability to directionally change the fundamental frequency of their vocalizations according to an auditory target. Based on previous research on VPL in birds and cetaceans [21–23], we developed a multi-stage training plan, which was used to train six adult pale spear-nosed bats to adjust their calls according to artificially modified auditory input, via an ultrasonic intercom.

## 2. Material and methods

Six adult male pale spear-nosed bats (*Phyllostomus discolor*) were trained for up to 4 h per day, 5 days per week from December 2017 until August 2018. Outside of the training periods, they were housed with 24 conspecifics. This experiment was approved by the German Regierung von Oberbayern (approval 55.2-1-54-2532-34-2015). The six bats were trained in separate boxes, which were described in detail previously [20]. All bats had participated in vocal conditioning experiments in these boxes before [20]. Each box was equipped with one ultrasound microphone, a photoelectric barrier, a light emitting diode (LED), a remote-controlled feeding device and a loudspeaker for stimulus playback (see the electronic supplementary material for details (table S1)).

### (a) Data acquisition

Data acquisition was controlled via a custom-written Matlab script. The experiment was split into two phases: in phase 1, the bats were each presented with six randomly chosen, unmodified playbacks of their own ‘typical’ calls that had been recorded in a previous experiment [20]. In phase 2, the same six template calls were presented downward-pitch-shifted by four semi-tones (electronic supplementary material, figure S1). The bats actively started a run by interrupting the photoelectric barrier, which activated the LED for up to 5 s, indicating the reactive state of the feeder. In both phases, a continuously recorded ring buffer (of 250 ms length; sampling rate: 192 kHz) was saved, if a vocalization exceeded the call level threshold (40 dB sound pressure level integrated over the total buffer size) within this 5 s interval. Specifically, when the photoelectric barrier was interrupted in phase 1, a single, randomly chosen playback of one of the six unmodified calls was started. If the bats then emitted a call that exceeded the call level threshold within the 5 s interval a food reward was triggered and the LED was switched off. In phase 2, the bats were presented with the downward-shifted versions of their own calls. In this second phase, a spectral low-pass criterion for the feeder trigger was activated, allowing only those calls that exceeded the call level threshold in a frequency range below the low-pass cut-off frequency to trigger the feeder. Even though not all calls emitted within the reactive 5 s interval triggered the feeder, all calls were still saved if they exceeded the call level threshold [24]. The low-pass, cut-off frequency was initially set to 27, 28 or 30 kHz and was then adjusted depending on the individual training success to final frequencies between 13.1 and 15.6 kHz (electronic supplementary material, figure S2). The six bats were recorded in phase 2 for varying durations (64–94 days, electronic supplementary material, figure S2), which allowed us to compare the datasets after 30 and after 60 days of training for all individuals.

In order to test whether the active low-pass criterion (indication for usage learning), or the presentation of frequency-shifted playbacks (indication for production learning) caused the observed change in mean *f_0_*, four bats were used for a follow-up experiment directly subsequent to their training in phase 2. Two bats (Bats 1 and 3) did not enter the follow-up experiment as they began data collection later than the other four. In the follow-up experiment, the low-pass criterion was deactivated for 5 days (the templates were still frequency-shifted (‘criterion deactivated’ data)), thus all calls exceeding the level threshold triggered a food reward. Subsequently, the unmodified ‘typical’ calls were used again as templates (‘unshifted template’ data) for a further 5 days. If the bats were following the template, we expected to see no change or even a further decrease in the *f*_0_ of their calls when the low-pass criterion was deactivated, but an increase in *f*_0_ when unshifted templates were presented.

### (b) Analysis

In the 250 ms long recordings, individual calls were automatically detected by a custom-written Matlab script, and call duration, level and mean *f*_0_ were extracted. To determine the *f*_0_ of a call, the YIN algorithm [25] was employed and detected *f*_0_ jumps were corrected for. From the trace of *f*_0_ over time, the mean *f*_0_ was calculated. In order to conservatively exclude echolocation calls from the analyses, only calls with a minimum duration of 5 ms were considered in the analyses. A correlation analysis was conducted on all calls of each individual to test for correlation between mean *f*_0_ and call duration. Owing to the low sample size, we conducted all statistical analyses within the individual subjects. In order to detect a change of *f*_0_ over time, we compared ‘baseline’ data with data ‘after 30 days of training’ and ‘after 60 days of training’. For these datasets, we pooled all calls from each bat separately over 5 days (i.e. data 5 days before phase 2 and from days 28–32 and 58–62 in phase 2). With the one-sample Kolmogorov–Smirnov test for continuous data, we confirmed that all our datasets differed significantly from normal distributions. Thus, we used the Wilcoxon rank-sum test to compare the datasets for each bat individually and report the number of analysed calls, median, interquartile ranges and *p*-values of the Wilcoxon rank-sum test [24].

## 3. Results

In the course of the experiment, 28 452 calls were recorded within the different datasets, each encompassing recordings from 5 days. The number of emitted calls increased when the low-pass filter was activated and decreased again after its deactivation (electronic supplementary material, tables S2 and S3). Together with a strong reduction in success rate whenever the low-pass filter was further lowered (electronic supplementary material, figure S2), this highlights the difficulty the low-pass filter presented to the bats.

**Figure 1.**
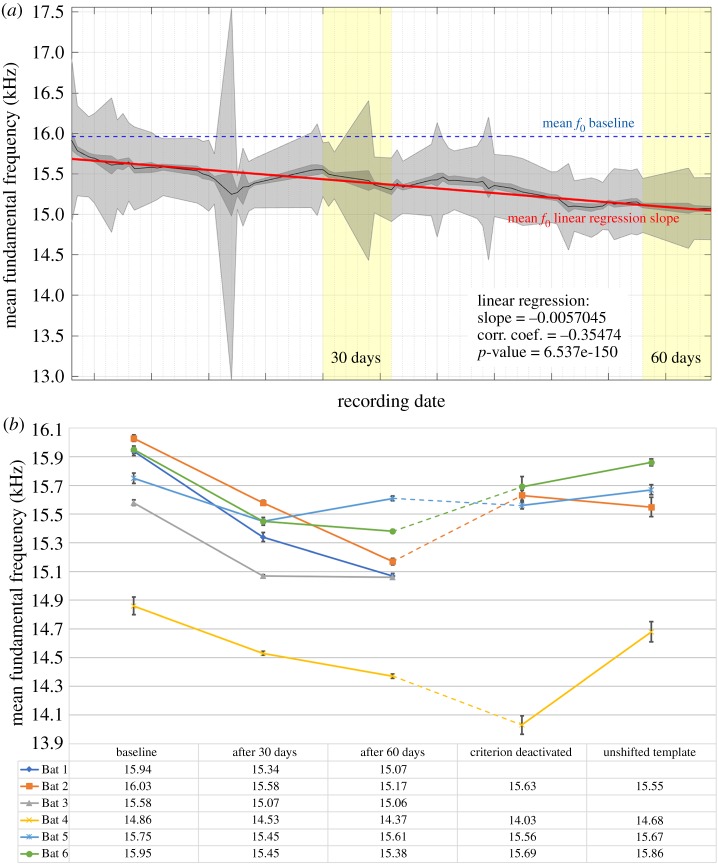
Change of mean fundamental frequency. (*a*) Exemplary trajectory of the *f*_0_ decrease of Bat 1. The data are displayed continuously over the first 62 days of acquisition and presented with a smoothed mean (bin: 5 days). The mean *f*_0_ of the baseline data (approx. 16 kHz, indicated by dotted blue line) and the actual mean *f*_0_ trajectory (solid black line) are shown. Standard error of the mean (s.e.m.) is indicated in dark grey and the standard deviation in light grey. The linear regression slope of the mean *f*_0_ is indicated with a solid red line. Yellow areas indicate the time points ‘30 days of training’ and ‘60 days of training’. (*b*) This shows the change of mean *f*_0_ of all six bats, pooled over 5 days in the different datasets. The different bats are represented by colours and grey error bars indicate the s.e.m. Bats 2 and 4–6 were tested in a follow-up experiment investigating their response to the deactivation of the low-pass filter criterion and the presentation of unshifted templates. These follow-up data were collected directly subsequent to training in phase 2 for all four bats. However, the duration of training in phase 2 differed between individuals (indicated by the dashed lines), details of which can be found in electronic supplementary material, figure S2.

All six bats significantly lowered the mean *f*_0_ of their calls within the first 30 days of training with pitch-shifted template calls (in each case *p* < 0.01, figure 1, electronic supplementary material table S2). Five of them further decreased their *f*_0_ within the next 30 training days, while one bat started to slightly increase the *f*_0_ of its calls again (Bat 5, figure 1*b*). However, after 60 days of training, the mean *f*_0_ of the calls of all six bats was significantly lower than in the ‘baseline’ data (average reduction of mean *f*_0_: 637 Hz, electronic supplementary material, table S2). All bats also significantly prolonged their calls in response to the training regime (electronic supplementary material, table S3). Mean *f*_0_ and call duration were negatively correlated for most bats (Bats 2–6), but slightly positively correlated for Bat 1 (electronic supplementary material, figure S3). Call level change was also noted in the different datasets; the maximal median level increase, however, was below 5 dB and lay within the variation arising from the bats' free movement in the set-up (electronic supplementary material, figure S4).

The individual bats reacted differently to the deactivation of the low-pass filter criterion and the presentation of an unshifted template. Two out of four bats (Bats 2 and 6) increased the *f*_0_ of their calls significantly after the low-pass criterion was deactivated (for both bats *p* *<* 0.01, electronic supplementary material, table S2). Neither of them significantly changed the *f*_0_ after the unshifted templates were played back (*p* = 0.78 (Bat 2) and *p* = 0.21 (Bat 6), figure 1*b*; electronic supplementary material, table S2). Another bat (Bat 5), did not show a significant change in pitch after the low-pass criterion was deactivated (*p* = 0.78), but significantly increased the *f*_0_ of its calls after unshifted templates were presented again (*p* < 0.01, figure 1*b*; electronic supplementary material, table S2). Bat 4 continued to decrease the *f*_0_ of its calls even after the low-pass filter criterion was deactivated and increased its *f*_0_ only after unshifted templates were presented (*p* < 0.01, figure 1*b*; electronic supplementary material, table S2).

## 4. Discussion

Using a set-up and training regime modified from the bird literature [21], we here demonstrate that *P. discolor* can directionally shift the fundamental frequency (*f*_0_) of their social calls. All six bats decreased the *f*_0_ of their calls significantly after 30 days of training, following playback of a pitch-shifted version of their own calls and a low-pass criterion for food reward. This decrease in *f*_0_ occurred gradually (figure 1*a*), suggesting that the *f*_0_ decrease is not a result of trial and error, but rather that it is guided by the presented pitch-shifted templates. The maximal observed decrease in mean *f*_0_ (990 Hz or 6.2%; electronic supplementary material, table S2) was a smaller pitch shift than the shift applied to the presented template call (24%). As the physiological range of pitch production for these specific calls is unknown, the limited decrease in mean *f*_0_ could be caused by limitations in the structural plasticity of their calls. Nevertheless, the pitch shift produced by all experimental animals is perceivable by this species (they can perceive a frequency change as little as 1% [26]), and the bats lowered the *f*_0_ of their calls actively and in a directional manner, demonstrating their capacity to directionally modify the spectral parameters of their vocalizations.

To test the driving force behind the decrease in *f*_0_, we first deactivated the low-pass criterion and only later presented unshifted templates. If the bats were driven by the template in order to adjust their *f*_0_, we would expect an increase in *f*_0_ only once the unshifted templates were presented (i.e. indication for VPL). If the bats were driven solely by the pressure exerted by the low-pass criterion, we would expect a prompt upward shift after the reward criterion was deactivated (i.e. indication for non-auditory learning). The results show that the experimental bats reacted differently in the training paradigm. Two out of four bats (Bats 2 and 6) started to increase the *f*_0_ of their calls when when the low-pass reward criterion was deactivated but the presented templates were still downwards-pitch-shifted (figure 1*b*). Bat 4, however, was driven by the pitch-shifted acoustic template and only increased its pitch after the unshifted templates were presented (figure 1*b*). This gradual vocal parameter change towards an acoustic target presents a case of limited VPL in this species. This was only shown clearly in a single animal, and as such this argues that the species has the capacity for this ability, even if it is not always employed. To further differentiate between vocal production and vocal usage learning, future experiments should also investigate these bats’ capacity for structural call imitation outside the normal species-specific repertoire. The ability to imitate such call templates would provide evidence for more complex VPL.

## 5. Conclusion

In this study, we demonstrate that isolated adult pale spear-nosed bats show the capacity for directional pitch shift of their vocalizations. Some bats were driven by the low-pass filter reward threshold instead of the playback, as two bats responded directly to the low-pass filter deactivation. However, one of the bats used auditory experience, rather than reward, to adjust the pitch of its calls, thus demonstrating limited VPL under strictly controlled experimental conditions. As for previous cases where individual animals demonstrated the extent of vocal learning in isolated individuals [23,27,28], this single case demonstrates the capacity of pale spear-nosed bats to perform limited VPL.

## Supplementary Material

Supplementary Material

## Data accessibility

All raw recordings and scripts used in this study can be found online and are accessible via the online repository G-Node: https://dx.doi.org/10.12751/g-node.d9dd71 [24].
