# Supplementary material for: Vocal production learning in the pale spear-nosed bat, Phyllostomus discolor
Source: Biol Lett. 2020 Apr 15;16(4):20190928. doi: 10.1098/rsbl.2019.0928 (PMC7211467; doi:10.1098/rsbl.2019.0928)
Supplement: Supplementary Material [file rsbl20190928supp1.docx]

**Supplementary material**

**Table S1: Equipment details of the setup.** The table presents the equipment used in the training and data acquisition setup, details the product information, and indicates the connections between the devices.

| **Equipment** | **Product information** |
| --- | --- |
| ultrasound microphone  (connected via the microphone preamplifier to the audio interface) | Custom-made on basis of SPU0410LR5H, Knowles Corporation, Itasca, IL, USA |
| microphone pre-amplifier  (level setting: -10dBV) | OctaMic II, RME, Haimhausen, Germany |
| multi-channel audio interface | Fireface 800, RME, Haimhausen, Germany |
| loudspeaker (tweeter)  (connected to audio interface via the power amplifier) | XT19NC30-04 Peerless, Tymphany HK Ltd., Sausalito, California, USA |
| power amplifier | Harman Kardon AVR 445, Garching, Germany |
| photoelectric barrier  (physical interruption stated an experimental run and activated the LED) | EE-SX461-P11 photomicrosensor, Omron Electronics, Langenfeld, Germany |
| **Software:** MATLAB  R2007b, v7.5.0.342  R2015a, v8.5.0.197613 | MathWorks, Cambridge, MA, USA  Data acquisition  Data analysis |

**Table S2: Change in the median of the mean *f_0_*.** The mean *f_0_* represents an average of the *f_0_* over the time course of each individual call. The table shows the comparison of the five data sets: ‘baseline’ (i.e. before the activation of the low-pass criterion and presentation of frequency-shifted templates), ‘after 30 days’ and ‘after 60 days’ of training, with the low-pass ‘criterion deactivated’, and presentation of ‘unshifted templates’. The difference between the data sets was tested using the Wilcoxon rank-sum test. For all data sets we report the number of analysed calls, median, interquartile ranges, and *p*-values.

*** = p-value < 0.0001; N = number of analysed calls (this excludes calls < 5 ms for Bats 1-5 and calls < 25 ms for Bat 6); IQR = interquartile range in kHz; diff. medians = difference between the medians of the two compared data sets (in kHz and %); ‘base line’ = data from 5 days before the activation of the low-pass criterion and the pitch-shifted template; ‘after 30 days of training’ = data from training days 28-32; ‘after 60 days of training’ = data from training days 58-62; ‘criterion deactivated’ = data from recording days 1-5 after the low-pass criterion was deactivated, but the template was still pitch-shifted; ‘unshifted template’ = data from recording days 6-10 after the low-pass criterion was deactivated, the template was not pitch-shifted anymore.

**Table S3: Change in median call duration.** The table shows the comparison of the five data sets: ‘baseline’ (i.e. before the activation of the low-pass criterion and presentation of frequency-shifted templates), ‘after 30 days’ and ‘after 60 days’ of training, with the low-pass ‘criterion deactivated’, and presentation of ‘unshifted templates’. The difference between the data sets was tested using the Wilcoxon rank-sum test. For all data sets we report the number of analysed calls, median, interquartile ranges, and *p*-values.

*** = p-value < 0.0001; N = number of analysed calls (this excludes calls < 5 ms for Bats 1-5 and calls < 25 ms for Bat 6); IQR = interquartile range in ms; diff. medians = difference between the medians of the two compared data sets (in ms and %); ‘base line’ = data from 5 days before the activation of the low-pass criterion and the pitch-shifted template; ‘after 30 days of training’ = data from training days 28-32; ‘after 60 days of training’ = data from training days 58-62; ‘criterion deactivated’ = data from recording days 1-5 after the low-pass criterion was deactivated, but the template was still pitch-shifted; ‘unshifted template’ = data from recording days 6-10 after the low-pass criterion was deactivated, the template was not pitch-shifted anymore.


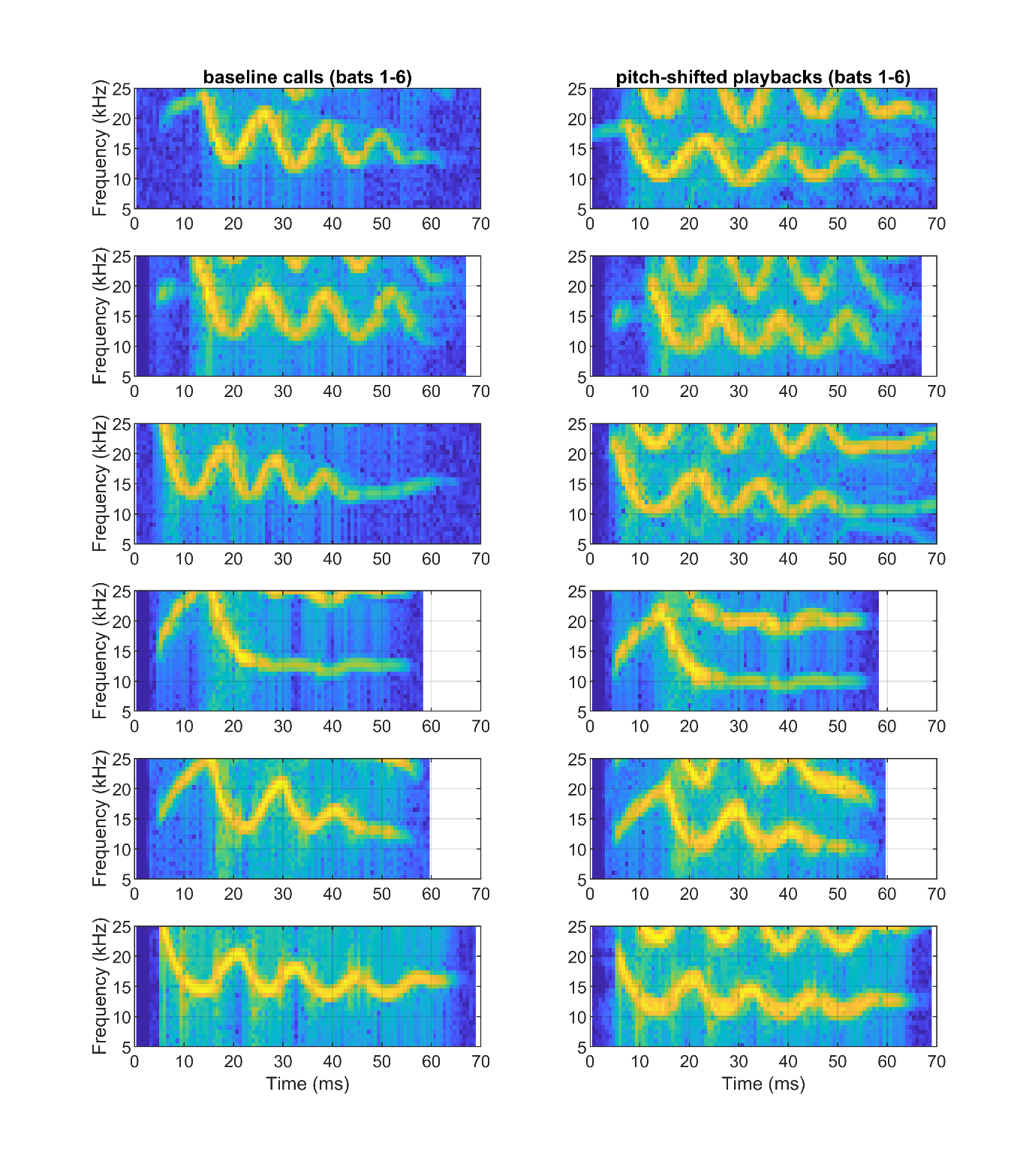


**Figure S1: Spectrograms of the typical baseline calls and the corresponding pitch-shifted template for each bat.** Top to bottom: Bats 1-6. The bats’ vocalisations all fall in the sinusoidally frequency modulated (SFM) syllable class described for this species (Lattenkamp *et al.*, 2019). The playback calls (right column) are downward pitch shifted by four semi-tones from the original frequency range in which they were recorded (left column). Specifically, a frequency-shift of four semi-tones equals a 24% change of the mean fundamental frequency of the calls (e.g. a call with a natural fundamental frequency (*f_0_*) of 15 kHz would be downward pitch-shifted by 3.6 kHz to a new fundamental frequency of 11.4 kHz).


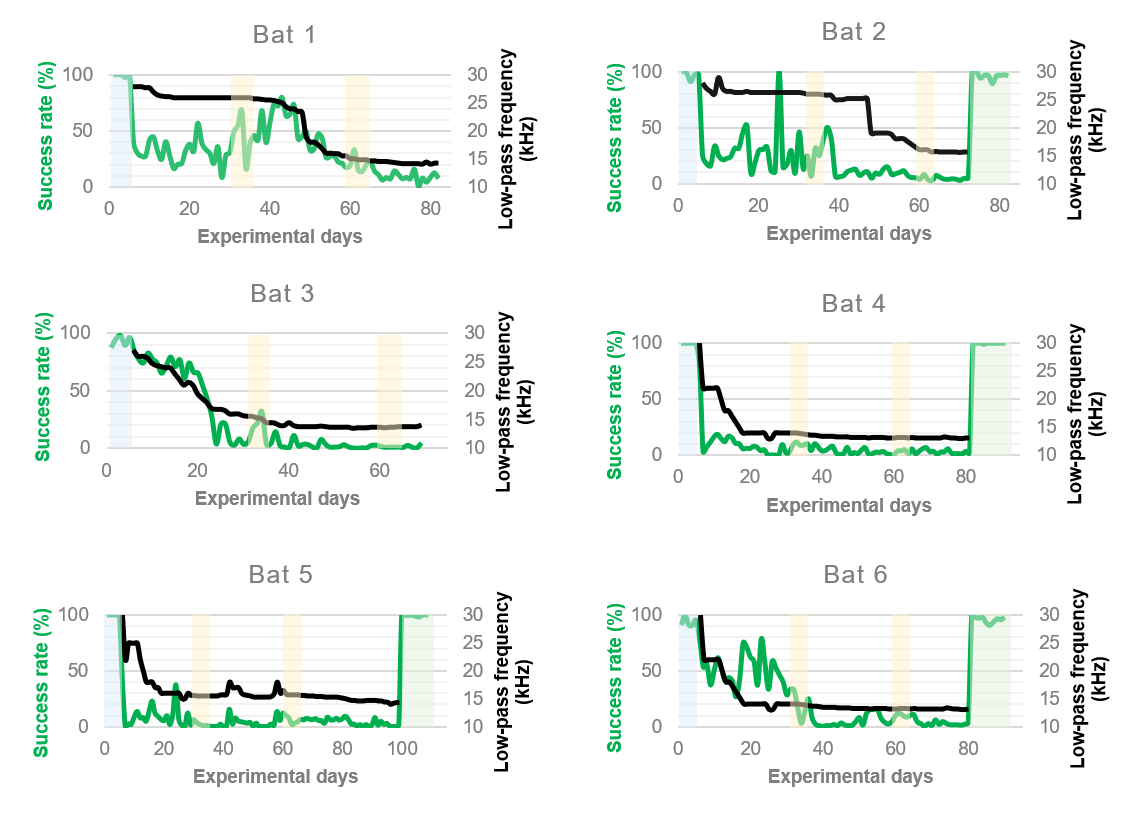


**Figure S2: Success rate in dependence of the individual low-pass filter settings.** In the presented contingency training paradigm, the level of difficulty was adjusted depending on the individual success rate in the experiment. The success rate (indicated in green) is calculated as the percentage of feeder activations in respect to the total number of saved recordings (i.e. all recordings that exceeded the level threshold). The low-pass cut-off frequency (indicated in black) was adjusted to maintain high pressure on the individuals, but at the same time ensure that a certain success rate could be reached daily in order to not demotivate the bats to participate in the experiment. The shaded areas show the analysed time points. Blue shading indicates the ‘baseline’ data, yellow indicates ’30 days’ and ’60 days’ of training, and green highlights the follow-up experiment (i.e. ‘criterion deactivated’ and ‘unshifted template’). One can see that the bats participated in training phase 2 for different time spans. However, the follow-up data was always collected directly subsequent to training phase 2. Notable is the immediate increase in success rate after the low-pass criterion was deactivated, indicating the strong pressure the low-pass criterion exerted over the bats. Bat 1 and Bat 3 did not participate in the follow-up data acquisition.

**
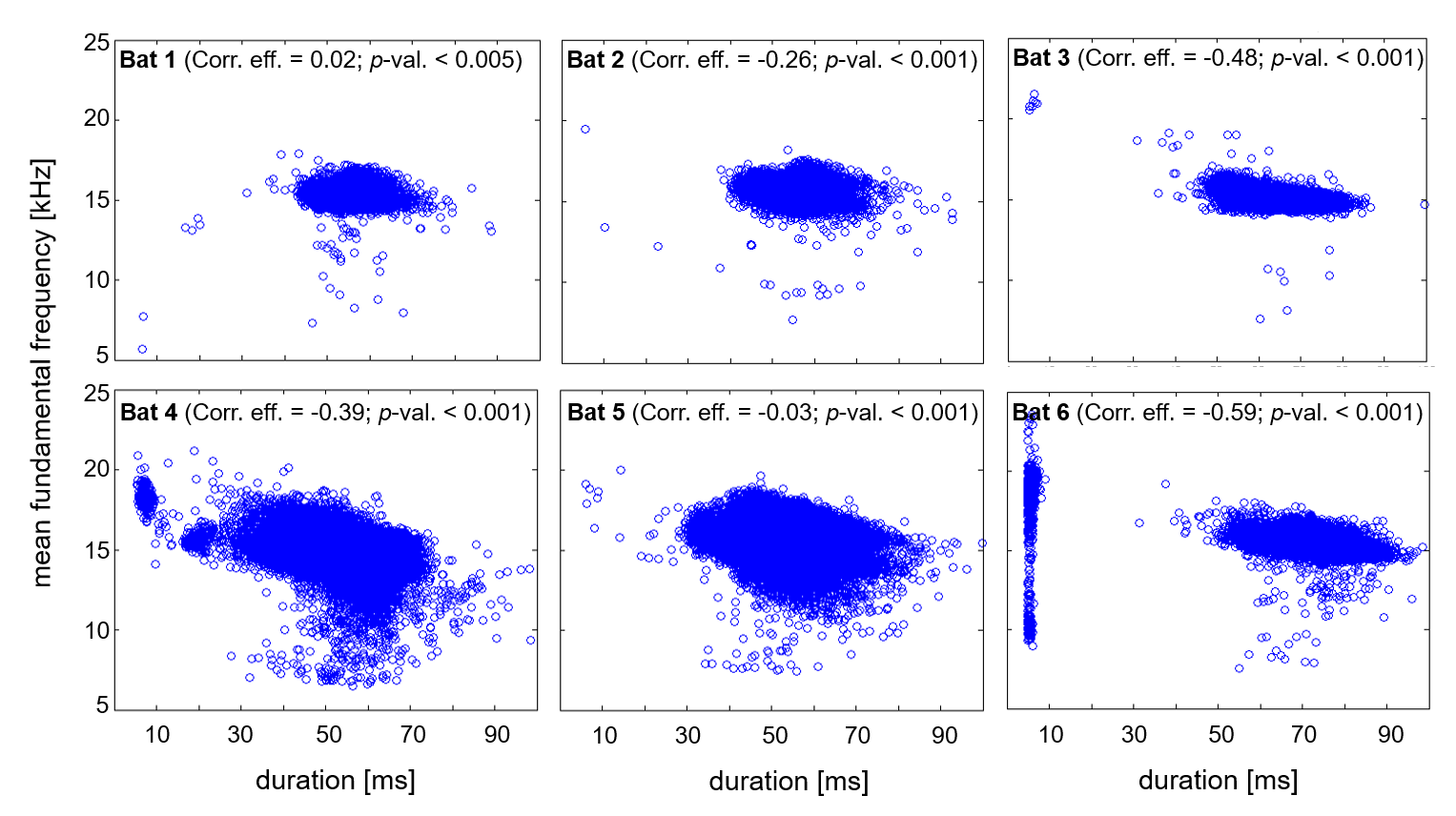
**

**Figure S3: Results of the correlation analysis of the mean *f_0_*** **and call duration.** Illustrated is a scatterplot of the pooled total calls of each individual. The correlation analysis demonstrates that *f_0_* can be negatively correlated with call duration (Bats 2-6), but also that this correlation is not imperative (Bat 1). Bat 6 used two call types, one of which was shorter and was more variable in its mean *f_0_*, however a negative correlation can be conceived within the longer calls of this individual. The prolongation of calls has been previously been reported as one of several coping strategies of these bats when faced with challenges in a vocal training paradigm (Lattenkamp *et al.*, 2018). Call prolongation was shown to be associated with pitch increase and decrease and can also occur independently from pitch change (Lattenkamp *et al*., 2018). Call duration and pitch are thus not intrinsically associated. A possible motivation for the call prolongation observed in this study, is that the vocalisations generally possess sinusoidally frequency modulated elements with a low *f_0_* tail (Fig. S1). If a call is lowered in its *f_0_* (i.e. in a way that the lowest points of the call are below the threshold), prolongation of the call would help to trigger the feeder as overall more energy is emitted in the lower frequency ranges.

**
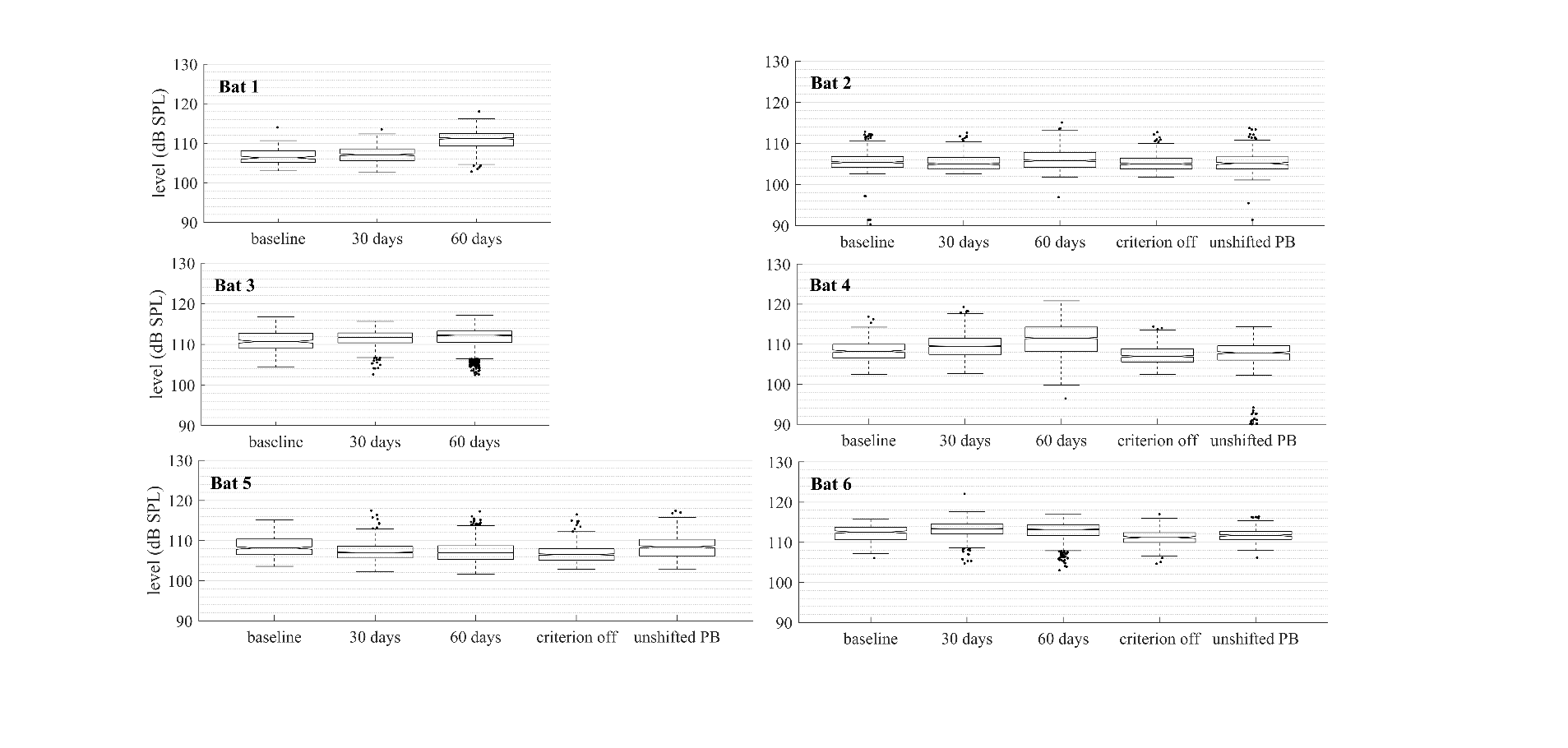
**

**Figure S4: Change in median call level emitted by bat 1-6.** The boxplots show the increase and decrease in call level recorded in the different data sets. Within the first 60 days of training call levels slightly increased for Bats 1-4, they decreased for Bat 5, and did not change for Bat 6. Even including the responses to the follow-up experiments, median call levels changed by less than 2 dB for Bats 2, 3, 5, and 6. Bat 1 exhibited the maximal change of median call level (4.7 dB [baseline vs 60 days of training]). While Bat 4 initially increased median call levels (2.8 dB [baseline vs 60 days of training]), it immediately decreased call levels after the low-pass criterion was deactivated (-3.6 dB [60 days of training vs criterion deactivated]). As the bats were free to move within the setup, the recorded call levels varied depending on the bats’ location in the setup. The potential call level variation due to orientation and positioning of the bats in the setup can result in amplitude differences of up to 6 dB. From our data, we cannot determine if the relatively small recorded call level differences were due to recording position of the bats or emission of louder calls.
